# Trifecta of Abscesses of the Prostate, Bilateral Seminal Vesicles, and Left Epididymis Caused by Staphylococcus aureus: A Report of a Rare Case

**DOI:** 10.7759/cureus.75573

**Published:** 2024-12-11

**Authors:** Makoto Nakamura, Masanori Matsukawa

**Affiliations:** 1 Department of Urology, Takikawa Municipal Hospital, Takikawa, JPN; 2 Department of Urology, Hakodate Goryoukaku Hospital, Hakodate, JPN

**Keywords:** abscess, epididymis, prostate, seminal vesicles, staphylococcus aureus

## Abstract

We report here a rare case of a concurrent occurrence of abscesses caused by *Staphylococcus aureus* in the prostate, seminal vesicles, and epididymis.

A 71-year-old male presented to our hospital with urinary retention, and an indwelling urethral catheter was inserted. He remained afebrile until a revisit one month later when he developed a fever and left scrotal swelling. Imaging studies revealed multiple abscesses in the left lobe of the prostate, bilateral seminal vesicles, and the left epididymis. Ceftriaxone was initiated upon admission and was administered for five days before switching to cefazolin for an additional 11 days. From the sixth day of admission, levofloxacin was added to ensure adequate prostatic tissue penetration until the 18th day. Owing to his poor response to antimicrobial chemotherapy, percutaneous prostatic needle aspiration, percutaneous cystostomy, transurethral deroofing of the abscess, and left orchiectomy were performed on the eighth day. Methicillin-susceptible *S. aureus* was isolated from the urine and abscess fluid. The patient had no recurrence at one year postoperatively.

A prostate abscess may not manifest as fever or elevated prostate-specific antigen levels, even when *S. aureus* is the causative agent, and can lead to delayed diagnosis and subsequent involvement of the seminal vesicles and epididymis.

## Introduction

The prostate gland is located at the crossroads between the urinary and male reproductive systems, plays an essential role in human reproduction, and is anatomically continuous with seminal vesicles and ductus deferens [1]. It serves as a focal point for bacterial infections, with acute prostatitis relatively common in clinical practice [2]. However, prostatic abscess (PA) formation is rare and can be severe or life-threatening [3,4]. The seminal vesicles that secrete a major part of the semen form the ejaculatory duct combined with the ductus deferens, which drains into the prostatic urethra [1]. Seminal vesicle abscess (SVA) is a rare pathological entity, with only a few reported cases [5]. The epididymis is connected to the prostate gland via ductus deferens [1]. Although epididymitis is commonly observed, abscesses of the epididymis (AE) are infrequent and occur as complications in cases of epididymitis [6].

The main causative organisms of PAs have shifted over time. In the pre-antibiotic era, *Neisseria gonorrhoeae* was the primary causative agent of PA, but since then it has been replaced by *Enterobacteriaceae*, most notably *Escherichia coli* [3,7]. Since the 2000s, *S. aureus*, particularly a methicillin-resistant strain, has been reported to be the predominant pathogen [7-9]. *S. aureus* is a rare cause of urinary tract infection in a community, with its prevalence ranging from 0.5% to 1% of positive urine cultures. *S. aureus* bacteriuria is prone to developing into potentially life-threatening invasive infections such as bacteremia [10].

We report a case of concurrent abscesses of the prostate, bilateral seminal vesicles, and left epididymis caused by methicillin-susceptible *Staphylococcus aureus* (MSSA).

## Case presentation

**Figure 1:**
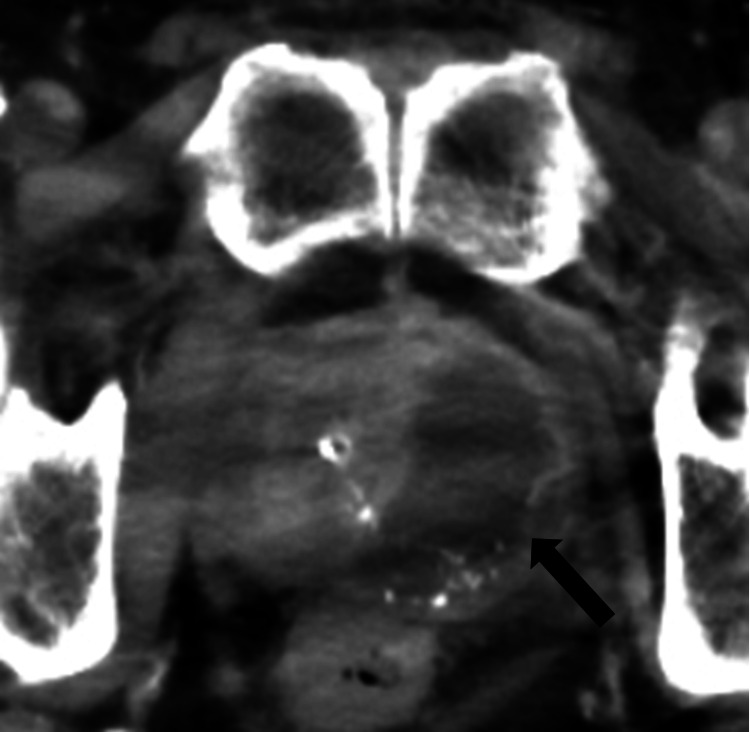
Pelvic non-contrast CT scan at the time of urinary retention A pelvic non-contrast CT scan at the time of urinary retention reveals a low-density area, 4.5 cm in diameter, located in the left lobe of the prostate (black arrow). The image was slightly blurred because of metal artifacts from the left total hip replacement. CT, computed tomography.

A 71-year-old male presented to the urology department with overflow urinary incontinence. He had been aware of difficult urination for several months, and two days before the initial visit, the symptom worsened and was accompanied by incontinence. His medical history included diabetes mellitus, hypertension, bronchial asthma, total replacement of the left hip joint, and cerebral infarction requiring an anticoagulant (clopidogrel sulfate) to prevent recurrence. He was afebrile and had not received any antimicrobial therapy prior to his initial visit. On clinical examination, the non-tender suprapubic mass was palpable. His digital rectal examination revealed an enlarged prostate without tenderness and induration. A urethral catheterization yielded 1450 ml of cloudy urine. A computed tomography (CT) scan revealed an enlarged prostate (estimated volume, 75 cm^3^) with a 4.5 cm low-density region in the left lobe (Figure 1). Blood analyses revealed a white blood cell (WBC) count of 15,350/μl and a prostate-specific antigen (PSA) level of 2.9 ng/ml. The patient was initiated on the alpha-blocker silodosin; however, he had a recurrence of urinary retention, necessitating the placement of an indwelling urethral catheter. Although MSSA was isolated from urine culture, antimicrobial chemotherapy was not administered due to the absence of fever, the normal level of PSA, and lack of awareness of the presence of an abscess.

**Figure 2:**
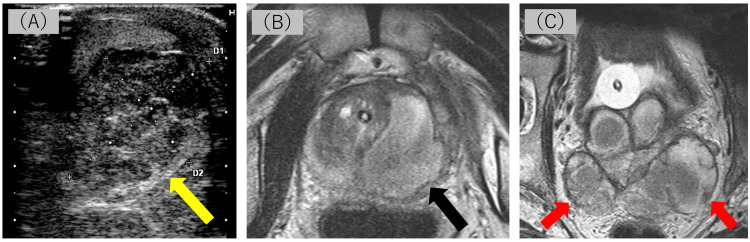
Ultrasonography and MRI at the time of hospital revisit. Scrotal ultrasonography shows a mosaic-like hypoechogenic area in the tail of the left epididymis (panel A). Pelvic T2-weighted MRI reveals fluid collection in the left lobe of the prostate and the left-dominant enlarged bilateral seminal vesicles (panels B and C). These images depict the simultaneous occurrence of abscesses of the prostate, bilateral seminal vesicles, and left epididymis. MRI, magnetic resonance imaging.

One month later, the patient returned to our hospital with a slight fever of 37.3 degrees Celsius, anorexia, left scrotal swelling, and skin redness that had begun two weeks earlier. A scrotal ultrasound revealed a 3.4 cm hypoechoic area with a mosaic pattern in the left epididymis (Figure 2, panel A). Pelvic magnetic resonance imaging (MRI) showed left-dominant enlargement of the bilateral seminal vesicles filled with fluid and a 4 cm PA in the left lobe (Figure 2, panels B and C).

**Figure 3:**
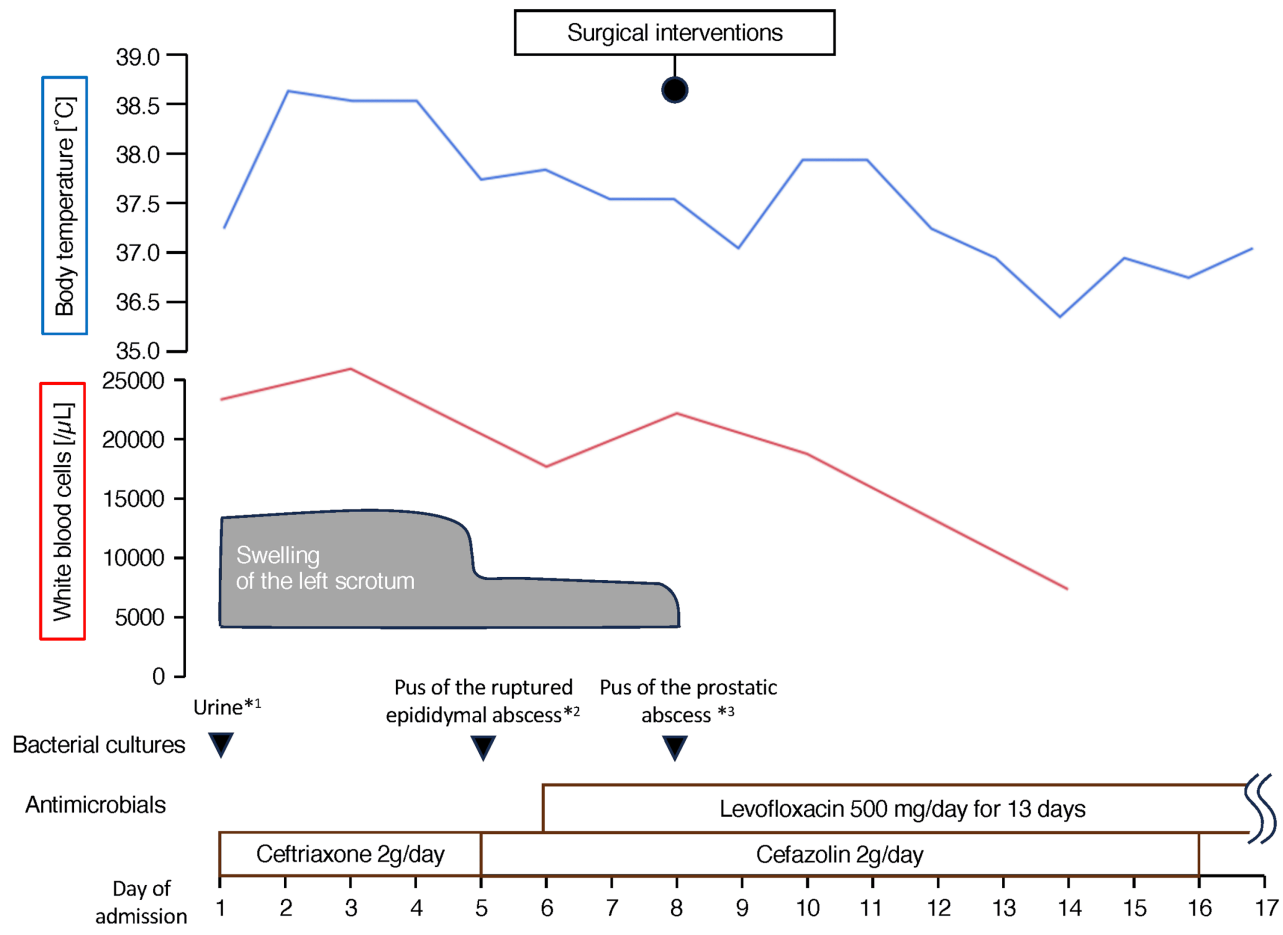
Schematic diagram of the clinical course after admission. *1, 3, *Staphylococcus aureus* was isolated. *2, *Staphylococcus aureus, Enterococcus faecalis, Bacteroides thetaiotaomicron*, and *Clostridium innocuum* were isolated.

Blood analyses revealed a WBC count of 23,560/μl and a C-reactive protein of 11.4 mg/dl. The patient was admitted to the hospital, ceftriaxone was initiated, and clopidogrel was discontinued (Figure 3). Blood cultures on admission were negative. The patient had persistent fever; however, his circulation stability and the absence of bacteremia allowed him to be on standby for surgical interventions involving a high risk of bleeding until the effect of clopidogrel diminished. Five days after admission, the epididymal abscess spontaneously ruptured, and purulent discharge was drained through the scrotal skin fistula (Figure 3). MSSA, *Enterococcus raffinosus*, *Bacteroides thetaiotaomicron*, and *Clostridium innocuum* were isolated from the pus from the fistula.

**Figure 4:**
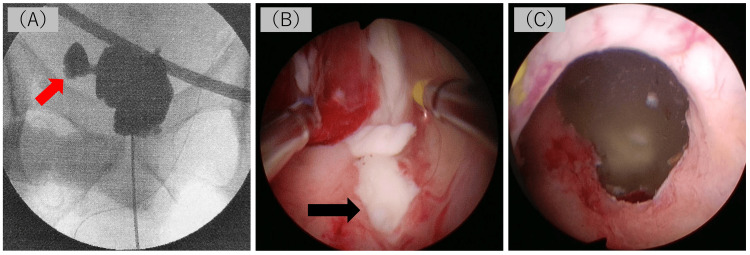
Intraoperative findings during transperineal abscess puncture and TU-deroofing of the prostate. Abscessography demonstrates communication between the cavity of the prostatic abscess and bilateral seminal vesicles (panel A). Emanation of the pus from the orifice of the left ejaculatory duct is observed under direct urethroscopic vision (black arrow, panel B). TU-deroofing of the abscess wall was performed sufficiently to drain the thick pus (panel C). TU-deroofing, transurethral deroofing.

Eight days after admission, we performed left orchiectomy combined with resection of the scrotal skin debridement, ultrasound-guided percutaneous prostatic needle aspiration with abscessography, percutaneous cystostomy, and transurethral deroofing (TU-deroofing) of the abscess. When the PA was punctured, highly viscous white pus was aspirated. Subsequent abscessography demonstrated communication between the cavity of the PA and the bilateral seminal vesicles (Figure 4, panel A). Therefore, direct drainage of seminal vesicles was not performed. Careful endoscopic inspection of the prostatic urethra revealed a purulent discharge from the orifice of the left ejaculatory duct (Figure 4, panel B). Subsequently, TU-deroofing was performed, widening the orifice by excising the abscess wall (Figure 4, panel C). 

MSSA was isolated from the pus of the PA. Five days after admission, ceftriaxone was switched to cefazolin as de-escalation according to the results of antimicrobial susceptibility testing and administered for 12 days in combination with levofloxacin, which is known for its good prostatic tissue penetration and can cover potential pathogens of EA other than MSSA (*E. raffinosus* and other bacteria) for 13 days starting on the sixth day of admission. Antimicrobial therapy was completed on the 18th day due to the absence of bacteremia, persistent fever, recurrence of scrotal swelling, and significantly decreased inflammatory markers. Although imaging studies were not performed at the time of antibiotic discontinuation, a follow-up CT scan obtained 21 days later confirmed the collapse of the PA and SVA cavities. He underwent transurethral resection of the prostate (TUR-P) to eliminate the need for cystostomy without infectious complications nine months after the discharge and remained healthy without recurrence of the abscesses one-year post-TUR-P.

## Discussion

The concurrent occurrence of a PA and abscesses in anatomically contiguous organs is a rare clinical presentation. To date, only three cases of PA with SVA (including spermatic cord abscess) [11-13], one case of PA with EA [14], and one case of EA with SVA [15] have been reported. However, a combination of concurrent PA, SVA, and EA has not been previously reported. Imaging studies such as ultrasonography, CT, and MRI are the preferred modalities to confirm the diagnosis of PA, SVA, and EA. The common sites of PA occurrence are the central and peripheral zones [4]. The principal treatment of these abscesses is antimicrobial chemotherapy and drainage [4,16]. 

Common risk factors for the development of PA, SVA, and EA include diabetes mellitus, immunosuppressive status, lower urinary tract obstruction (especially urinary retention), urological procedures, and previous urinary tract infections [16]. Nearly all of the factors, which also overlap those for urinary tract infections caused by *S. aureus* [10], were present in the patient in this case. In addition, elevated inflammatory markers, urinary retention, and a cystic lesion in the left lobe of the prostate on CT at the initial visit suggested a PA. However, the absence of fever, pain, and normal PSA level led to delayed diagnosis. Blood cultures obtained at the time of admission were negative, indicating that the *S. aureus* bacteriuria was not secondary to bacteremia. No fever or pain associated with abscess formation was observed until the epididymis was involved. The prolonged period before the initiation of antimicrobial therapy may have facilitated the involvement of the seminal vesicles, as demonstrated by abscessography, and the left epididymis via the vas deferens. The absence of fever in PA has been reported in 40-50% of cases, even when caused by *S. aureus* [9,16]. Initial symptoms of PA with urinary retention, as in this case, were observed in 27.5% of patients and in 49% of patients without concomitant bacteremia [9]. Unlike acute prostatitis, where all cases show an increase in PSA levels [17], there are reports of two of seven cases of PA without an increase in PSA levels [18]. Cases of PAs caused by *S. aureus* without fever have been reported in the literature [9], but the underlying mechanism has not been elucidated. In this case, pus drainage from the ejaculatory duct orifice, albeit a small amount, may have reduced tissue destruction, partially explaining the absence of fever and PSA elevation.

The standard antimicrobial agents for *S. aureus* infections are nafcillin, oxacillin, and cefazolin for MSSA and vancomycin for MRSA [19]. On the other hand, fluoroquinolones such as levofloxacin are recommended to treat infections of the prostate and seminal vesicles because of their excellent penetration into those organs [20]. The duration of antibiotic therapy for PAs is not standardized and is influenced by factors such as the location and extent of the abscess, the presence or absence of surgical drainage, and the patient's response to treatments. In this case, transurethral deroofing resulted in the drainage of not only the PA but also the communicating SVA, obviating the need for direct drainage of the seminal vesicles. Additionally, excision of the scrotal content in combination with the skin fistula and vas deference up to the level of the internal inguinal ring to remove the infected lesion to the degree feasible may have contributed to the favorable treatment response.

## Conclusions

To the best of our knowledge, this is the first reported case of concurrent abscesses in the prostate, seminal vesicles, and epididymis. Even when *S. aureus* is the causative pathogen, PA may present without fever or elevated PSA levels. However, the mechanism underlying the absence of fever remains unclear. If the diagnosis is delayed, PA can progress and spread to seminal vesicles and epididymis.
